# Molecular Detection of Insecticide Resistance Mutations in *Anopheles gambiae* from Sierra Leone Using Multiplex SNaPshot and Sequencing

**DOI:** 10.3389/fcimb.2021.666469

**Published:** 2021-08-19

**Authors:** Jianhai Yin, Frederick Yamba, Canjun Zheng, Shuisen Zhou, Samuel Juana Smith, Lili Wang, Hongmei Li, Zhigui Xia, Ning Xiao

**Affiliations:** ^1^National Institute of Parasitic Diseases, Chinese Center for Disease Control and Prevention; Chinese Center for Tropical Diseases Research; WHO Collaborating Centre for Tropical Diseases; National Center for International Research on Tropical Diseases, Ministry of Science and Technology; Key Laboratory of Parasite and Vector Biology, Ministry of Health, Shanghai, China; ^2^National Malaria Control Program, Ministry of Health and Sanitation, Freetown, Sierra Leone; ^3^Division of Infectious Diseases, Chinese Center for Disease Control and Prevention, Beijing, China; ^4^Center for Global Public Health, Chinese Center for Disease Control and Prevention, Beijing, China

**Keywords:** Multiple insecticide resistance, *Anopheles gambiae*, *kdr*, SNaPshot, Sierra Leone, *rdl*, *ace-1*

## Abstract

Vector control interventions including long-lasting insecticidal nets and indoor residual spraying are important for malaria control and elimination. And effectiveness of these interventions depends entirely on the high level of susceptibility of malaria vectors to insecticides. However, the insecticide resistance in majority of mosquito vector species across African countries is a serious threat to the success of vector control efforts with the extensive use of insecticides, while no data on insecticide resistance was reported from Sierra Leone in the past decade. In the present study, the polymerase chain reaction was applied for the identification of species of 757 dry adult female *Anopheles gambiae* mosquitoes reared from larvae collected from four districts in Sierra Leone during May and June 2018. And the mutations of *kdr*, *rdl*, *ace-1* genes in *An. gambiae* were detected using SNaPshot and sequencing. As a result, one sample from Western Area Rural district belonged to *Anopheles melas*, and 748 *An. gambiae* were identified. Furthermore, the *rdl* mutations, *kdr* west mutations and *ace-1* mutation were found. The overall frequency was 35.7%, 0.3%, 97.6% and 4.5% in A296G *rdl*, A296S *rdl*, *kdrW* and *ace-1*, respectively. The frequencies of A296G *rdl* mutation (*P* < 0.001), *kdrW* mutation (*P* = 0.001) and *ace-1* mutation (*P* < 0.001) were unevenly distributed in four districts, respectively, while no statistical significance was found in A296S *rdl* mutation (*P* = 0.868). In addition, multiple resistance patterns were also found. In conclusion, multiple mutations involved in insecticide resistance in *An. gambiae* populations in Sierra Leone were detected in the *kdrW*, A296G *rdl* and *ace-1* alleles in the present study. It is necessary to monitor vector susceptibility levels to insecticides used in this country, and update the insecticide resistance monitoring and management strategy.

## Introduction

Malaria is one of the most widespread infectious diseases globally, with a major burden causing the death of more than 400,000 people yearly in the past three years, most of them were reported in the WHO African Region and children aged under five years (WHO, 2018; WHO, 2019; WHO, 2020). Sierra Leone is an area of stable malaria endemicity. The high malaria disease burden in this country accounted for approximately 48% of outpatient morbidity and approximately 38% of mortality in children under five years according to the national Malaria Indicator Survey 2016 (National Malaria Control Programme (NMCP) et al., 2016).

Vector control is an essential component of malaria control and elimination. At present, long-lasting insecticidal nets and indoor residual spraying are still two core interventions for malaria vector control. The effectiveness of these interventions depends entirely on the high level of susceptibility of malaria vectors to the insecticides. However, the insecticide resistance in majority of mosquito vector species across African countries is a serious threat to the success of vector control efforts with the extensive use of insecticides (https://anopheles.irmapper.com/).

Target-site resistance is one of the most main mechanisms of insecticide resistance. Alterations in the target sites can reduce sensitivity of the target receptors to insecticide. Four main chemical classes (organochlorines, organophosphates, carbamates, and pyrethroids) are used in the vector control, and only pyrethroids are for use on nets. Moreover, mutations in the *ace-1* gene encoding the target site of acetylcholinesterase (AChE) for organophosphate and carbamate insecticides (Weill et al., 2003; Weill et al., 2004; Fournier, 2005; Djogbenou et al., 2008), the *rdl* (resistance to dieldrin) gene (Ffrench-Constant, 1994; Du et al., 2005), and the amino acid sequence in the voltage-gated sodium channel referred to as knockdown resistance (*kdr*) (resistance to DDT and pyrethroids) (Martinez Torres et al., 1998; Ranson et al., 2000; Davies et al., 2007; Donnelly et al., 2009), have been found in *Anopheles gambiae*, which is a main malaria vector in Africa. For example, pyrethroid resistance is high in *An. gambiae* in West Africa including Benin, Burkina Faso, Ghana, Mali, Nigeria, etc. (Vincent and N’Guessan, 2013), while no data was reported from Sierra Leone in the past decade.

Moreover, strategies for insecticide resistance management in vector control must be implemented on the basis of local epidemiological and entomological data. Hence, bioassays using WHO defined diagnostic doses of insecticide, dose response bioassays, biochemical assays to detect activity of enzymes associated with insecticide resistance, and molecular assays to detect known resistance alleles, have been developed to facilitate the implementation of vector control interventions (Ranson et al., 2011; Vincent and N’Guessan, 2013). Among them, the molecular assays are very sensitive, and they can detect resistant alleles at low frequencies at an early stage of resistance development, which, therefore, provide an early warning of future resistance.

In addition, the World Health Organization (WHO) supported the Sierra Leone National Malaria Control Program (NMCP) to develop an Insecticide Resistance Monitoring and Management Program (IRMMP) 2017-2020 to conduct insecticide resistance monitoring in the four pilot districts - Bo, Bombali, Kono and Western Area Rural district, in order to maintain the effectiveness of existing insecticidal vector control interventions, adhering to the existing malaria strategic plan and linking with other specific implementation documents of the NMCP and the Ministry of Health and Sanitation (MOHS), Sierra Leone.

In this study, molecular detection of mutations in the three genes of *ace-1*, *rdl* and *kdr* were performed to understand the insecticide resistance of the female *An. gambiae* to the four classes of insecticides recommended by WHO for vector control especially the pyrethroids in Sierra Leone.

## Materials and Methods

### Sample Source and PCR-Species Identification

Adult female *Anopheles gambiae* mosquitoes were stored in the 1.5 ml Eppendorf tubes with cottons and silico gels (one mosquito per tube). They were reared from larvae, which were collected from natural breeding water bodies in Kayangba community from Bo district, Magbema community from Bombali district, Lebanon community from Kono district, and Bolima community from Western Area Rural district, respectively, during May and June 2018. And these four districts were selected into a four-year insecticide resistance monitoring and management program (2017-2020) which was supported by WHO to the National Malaria Control Program, Sierra Leone. Upon collection, larvae were kept in separate labelled buckets, transported to the insectary and maintained under optimal condition for mosquitoes rearing.

All adult females used for molecular characterization were morphologically identified as belonging to the *An. gambiae* complex. Genomic DNA was extracted from one mosquito respectively using the Qiagen DNA Mini Kit (Qiagen, Germany) according to the manufacturer’s instruction. The identification of single specimens of the *An. gambiae* complex was performed by a PCR (Scott et al., 1993).

### Multiplex PCR Amplification of Multiple Insecticide Resistance Genes

Three pairs of primers to detect *kdr* west and *kdr* east mutation, *rdl* Ala296Gly and *rdl* Ala296Ser mutation, and *ace-1* G119S mutation were used (**Table 1**) (Bass et al., 2007; Bass et al., 2015a; Bass et al., 2015b). Multiplex PCR was performed in a volume of 20 μl containing 1x GC-I buffer (Takara, Japan), 3.0 mM Mg2+ (Takara, Japan), 0.3 mM dNTP (Generay Biotech, Shanghai), 1 U Hot Star Taq polymerase (Qiagen, Germany), 1 µl of template DNA and 1 µl of primers (Sangon Biotech, Shanghai) (**Table 1**). Thermal cycler conditions were: 95°C for 2 min; 11 cycles of 94°C for 20 s, 65°C for 40 s, and 72°C for 1.5 min; 24 cycles of 94°C for 20 s, 59°C for 30 s, and 72°C for 1.5 min; and finally 2 min at 72°C. Multiplex PCR products were separated by electrophoresis on 2% agarose gels stained with ethidium bromide (Sangon Biotech, Shanghai). The remaining PCR products were purified with 5 units of shrimp alkaline phosphatase (SAP) (Promega, USA) and 2 units of exonuclease I (EXO I) (Epicentre, USA) at 37°C for 1 h and 75°C for 15 min, to remove excess dNTPs and primers, respectively.

**Table 1:** Multiplex PCR primers for detection of multiple insecticide resistance genes in *An. gambiae*.

Locus	Primer	Sequence (5´→3´)	Concentration (µM)	PCR Length (bp)	Reference
*ace-1*	ace-1-F	GGCCGTCATGCTGTGGAT	1.5	50	Bass et al., 2015a
	ace-1-R	TGGCGGTGCCGGAGTAGA			
*kdrE*/*kdrW*	kdr-F	CATTTTTCTTGGCCACTGTAGTGAT	1	71	Bass et al., 2007
	kdr-R	CGATCTTGGTCCATGTTAATTTGCA			
A296S/A296G	rdl-F	TCATATCGTGGGTATCATTTTGGCT	1	98	
	rdl-R	CGACATCAGTGTTGTCATTGTCAAG			Bass et al., 2015b

### SNaPshot Single Nucleotide Extension Reaction Assay and Sequencing

SNaPshot extension primers for detection of *kdr* west (L1014F) and *kdr* east (L1014S) mutations, A296G *rdl* (Ala-Gly) and A296S *rdl* (A296S) mutations, and G119S *ace-1* (Gly-Ser) mutation were designed (**Table 2**) to anneal on the sense strand immediately adjacent to the mutation site. Each extension primer was synthesized with a different length of poly (dT) tail to allow separation of SNaPshot products on the basis of size. SNaPshot analysis was performed using an Applied Biosystems SNaPshot Multiplex Kit (Applied Biosystems Co., Ltd., USA). Extension reactions were performed in a volume of 10 μl containing 5 μl of SNaPshot Ready Multiplex Ready Reaction Mix, 1 μl of extension primer mix (**Table 2**) and 2 μl of ddH_2_o. Thermal cycler conditions were: 96°C for 1 min; 28 cycles of 96°C for 10 s, 50°C for 5 s, and 60°C for 30 s. Each 10 μl of extension products were purified with 1 unit of SAP at 37°C for 1 h and 75°C for 15 min. Then, 0.5 μl of the purified extension products were mixed with 0.5 μl of Liz120 size standard, and 9 μl of Hi-Di™ formamide, and were denatured at 95°C for 5 minutes then sequenced using ABI3730XL. Analysis was performed using GeneMapper 4.1 (Applied Biosystems Co., Ltd., USA).

**Table 2:** SNaPshot extension primers for the detection of multiple insecticide resistance genes mutations in *An. gambiae*.

Locus	Probe	Sequence (5´→3´)	Concentration (µM)
*ace-1*	ace-1-SR	TTTTCGGTGCCGGAGTAGAAGC	1.6
*kdrE*	kdrE-SF	TTTTTTTGGCCACTGTAGTGATAGGAAATT	0.8
A296G	A296G-SR	TTTTTTTTTTTTCATTGTCAAGACAGTAGTTACACCTAAT	0.8
*kdrW*	kdrW-SR	TTTTTTTTTTTTTTTTTTCCATGTTAATTTGCATTACTTACGAC	0.8
A296S	A296S-SF	TTTTTTTTTTTTTTTTTTTTTTTTTTTTAAATGCTACACCAGCACGTGTT	1.6

### Data Statistics

Data was entered in Microsoft Excel 2010 and analyzed using IBM SPSS Statistic 20. Frequency counts for mutations of insecticide resistance genes in mosquitoes from different districts were compared using Pearson Chi-Square test or Fisher’s exact test performed at 0.05 level of significance.

## Results

### Sample Composition

A total of 757 adult anopheline mosquitoes reared from larvae were analyzed in the present study. In detail, 271, 253, 112, and 121 mosquitoes were from Kayangba community, Magbema community, Lebanon community, and Bolima community, respectively.

Moreover, a total of 8 samples from Kayangba (1), Magbema (1), and Bolima (6) were detected as negative results of the *An. gambiae* complex by PCR (Scott et al., 1993), and one sample belonged to *Anopheles melas*, the remaining 748 samples were *An. gambiae* (**Table 3**).

**Table 3:** PCR authentication for the members of the *An. gambiae* complex.

Community	*An. gambiae*	*An. melas*	Negative	Total
**Kayangba (Bo)**	270	0	1	271
**Magbema (Bombali)**	252	0	1	253
**Lebanon (Kono)**	112	0	0	112
**Bolima (Western Area Rural)**	114	1	6	121
**Total**	748	1	8	757

### Mutations of Multiple Insecticide Resistance Genes

**Figure 1:**
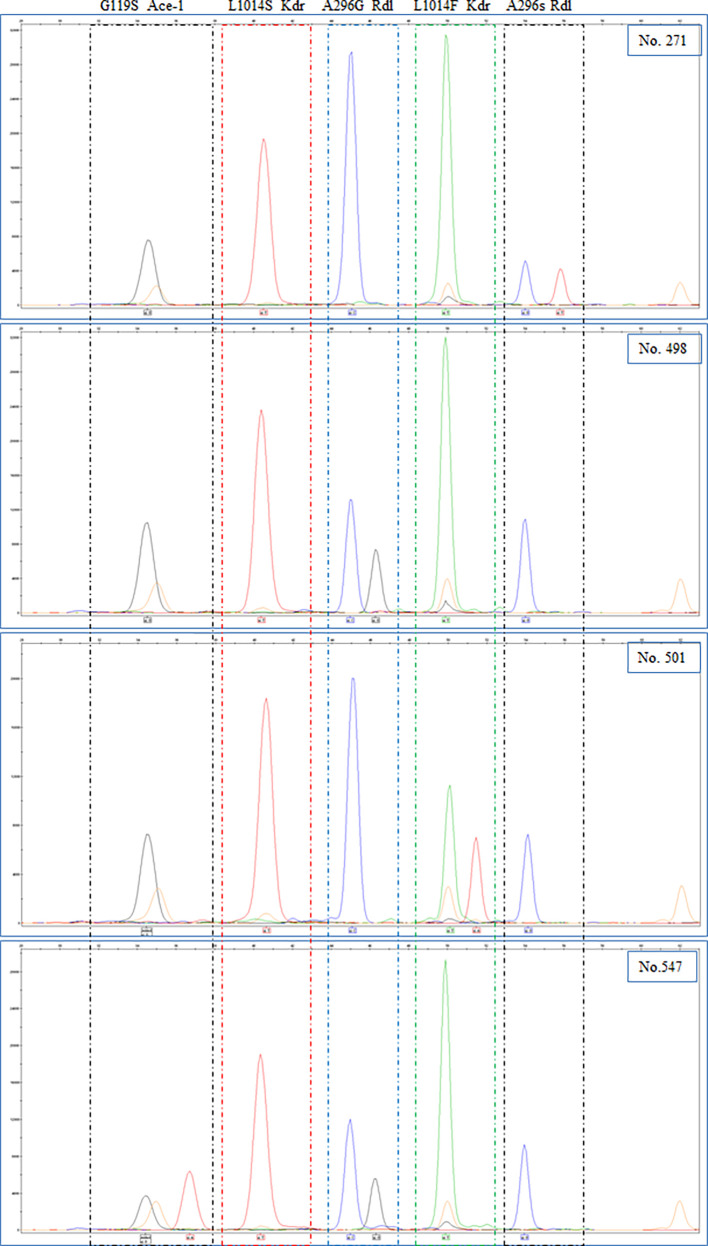
Electropherograms of genscan analysis of the SNaPshot reaction and alleles were indicated at the bottom. (Blue peak: G; Black peak: C; Green peak: A; Red peak: T; Orange peak: size standard).

A total of 748 adult *An. gambiae* were analyzed for presence of the *rdl* mutations, *kdr* mutations and G119S *ace-1* mutation (**Table 4**). A296G *rdl* mutation, *kdrW* mutation and *ace-1* mutation were identified at all 4 geographical sites, while A296S *rdl* mutation was only found in the samples from Kayangba and Magbema communities, and no *kdrE* mutation was found at all 4 sites. The overall mutation frequency was 35.7%, 0.3%, 0.0%, 97.6% and 4.5% in A296G *rdl*, A296S *rdl*, *kdrE*, *kdrW* and *ace-1*, respectively (**Table 4**). Moreover, the frequencies of A296G *rdl* mutation (χ^2^ = 377.148, *df* = 3, *P* < 0.001), *kdrW* mutation (Fisher’s exact test, *P* = 0.001) and *ace-1* mutation (χ^2^ = 42.989, *df* = 3, *P* < 0.001) were significantly different among four districts, respectively, while no statistical significance was found in A296S *rdl* mutation (Fisher’s exact test, *P* = 0.868). In addition, there were 12 types of allelic combinations totally including the sites that test failed, and a simple homozygous resistant mutation of *kdrW* (319), or combined homozygous resistant mutation of A296G *rdl* (174), or combined heterozygous mutation of A296G *rdl* (154), were the three most common combinations (**Table 5**). And four samples are provided as examples to show the results of sequencing (**Figure 1**).

**Table 4:** Genotypes of multiple insecticide resistance genes and their mutation frequency in the *An. gambiae*.

Community	A296G			F	A296S			F	*kdrE*				F	*kdrW*				F	*ace-1*				F
	RR	RS	SS		RR	RS	SS		RR	RS	SS	Failed		RR	RS	SS	Failed		RR	RS	SS	Failed	
**Kayangba**	178	81	11	0.809	0	1	269	0.002	0	0	270	0	0.000	270	0	0	0	1.000	0	7	263	0	0.013
**Magbema**	0	38	214	0.075	0	3	249	0.006	0	0	250	2#	0.000	227	23	0	2#	0.946	0	7	244	1#	0.014
**Lebanon**	1	28	83	0.134	0	0	112	0.000	0	0	112	0	0.000	108	4	0	0	0.982	0	20	92		0.089
**Bolima**	4	21	89	0.127	0	0	114	0.000	0	0	112	2*	0.000	111	1	0	2*	0.978	2	29	82	1*	0.145
**Total**	183	168	397	0.357	0	4	744	0.003	0	0	744	4	0.000	716	28	0	4	0.976	2	63	681	2	0.045

RR, homozygous resistant; RS, heterozygous; SS, homozygous susceptible; F, frequency.

# and * indicates that the failed samples are the same samples.

**Table 5:** The mutations of the multiple insecticide resistance genes in *An. gambiae* from communities.

Allelic combination					Number of mosquitoes detected				Total
A296G *rdl*	A296S *rdl*	*kdrE*	*kdrW*	G119S *ace-1*	Kayangba	Magbema	Lebanon	Bolima	
C/C	G/G	T/T	T/T	G/G	10	181	68	60	319
C/C	G/G	T/T	T/T	G/A	0	5	11	25	41
C/C	G/G	T/T	T/A	G/G	0	23	4	1	28
C/C	G/G	T/T	T/T	A/A	0	0	0	1	1
C/C	G/T	T/T	T/T	G/G	1	3	0	0	4
C/C	G/G	Failed	Failed	Failed	0	1	0	1	2
C/C	G/G	Failed	Failed	G/G	0	1	0	1	2
C/G	G/G	T/T	T/T	G/A	1	2	8	3	14
C/G	G/G	T/T	T/T	G/G	80	36	20	18	154
G/G	G/G	T/T	T/T	G/A	6	0	1	1	8
G/G	G/G	T/T	T/T	G/G	172	0	0	2	174
G/G	G/G	T/T	T/T	A/A	0	0	0	1	1

## Discussion

This study has analyzed the species composition in the *An. gambiae* complex and the mutations of the multiple insecticide resistance genes in *An. gambiae* in Sierra Leone, 2018. Adult female mosquitoes were reared from larvae, which were collected from several identified water bodies in four pilot districts for insecticide resistance monitoring in Sierra Leone. And only *An. gambiae* (99.9%, 748/749) and *An. melas* (0.1%, 1/749) were characterized in the present study, which is consistent with Sierra Leone’s records on the species distribution of *An. gambiae* complex (National Malaria Control Programme, 2015).

Key to the success of malaria control and elimination is a strategic plan on malaria vector control informed by comprehensive monitoring and evaluation of insecticide resistance, this is becoming more important as insecticide resistance increases and spreads across Africa, but there was absence of insecticide resistance monitoring and entomological work in general in the presence of large scale use of insecticides for malaria control in Sierra Leone. Therefore, it is necessary to carry out work in this area to promote the malaria control and elimination in this country.

Currently most resistance monitoring is dependent on bioassays which detect resistance under defined insecticide concentrations and exposure times recommended by WHO, but these methods require large numbers of alive mosquitoes and lack sensitivity (Ranson et al., 2011; Vincent and N’Guessan, 2013). And several alternative methods for detecting resistance have been developed, especially molecular tests based on DNA providing an early warning of emergence of the resistance are available for target sites with high sensitivity, although they are just a complement rather than a substitute for bioassays. And PCR or an intentional mismatched primer - PCR (IMP - PCR) followed by agarose gel electrophresis, or real - time TaqMan assays have typically been applied to the characterization of allelic mutations of the single insecticide resistance genes, respectively (Bass et al., 2010; Donnelly et al., 2016; Mavridis et al., 2018). Although reliable, these are time consuming with large numbers of samples for detection of multiple resistance mutations. The SNaPshot method used in the present study offers a specific, sensitive, inexpensive and rapid alternative to the simultaneous screening for multiple mutations and subsequent confirmation by sequencing (Quintans et al., 2004; Alina et al., 2005; Fondevila et al., 2017). This method has been extensively used in the blood group typing (Palacajornsuk et al., 2009; Latini et al., 2014; Chen et al., 2019) and common mutations in genes related to some cancers (Filippini et al., 2007; Hurst et al., 2009; Fariña Sarasqueta et al., 2011).

With regard to the frequency of mutations, a high mutation frequency of *kdrW* (97.6%) was found totally and most of them were homozygous (**Table 4**), and distributed in all four pilot sites. It supports the findings that pyrethroid resistance is predominant in *An. gambiae s.s.* in West Africa (Vincent and N’Guessan, 2013). Moreover, the *kdrW* mutation combined with one to two mutations at other three sites (A296G *rdl*, A296S *rdl*, *ace-1*) were found (**Table 5**), it revealed for the first time in Sierra Leone to our knowledge that multiple resistance patterns existed. However, standardized bioassays are still needed to test the resistance phenotype in individuals and combined with the molecular tests, to understand the real resistance status in this country. For example, there was a report that *An. gambiae s.l.* with the L1014F mutation were still susceptible to pyrethroids in Guinea Bissau (Dabire et al., 2008). Meanwhile, the failure to detect allelic mutations by molecular tests also cannot be interpreted as an absence of resistance in a population, and it reminds us that the detection system used in the present study should be further optimized. In addition, resistance mutations in different genes of A296G *rdl*, *kdrW* and *ace-1* were unevenly distributed in four pilot sites (**Table 4**). The mutation in A296G *rdl* (80.9%) was more frequent in samples from Kayangba community than other three communities. And no mutation was detected in A296S *rdl* in samples from Lebanon and Bolima communities, and very few samples were heterozygous individuals in the other two sites. Furthermore, the mutation in *ace-1* was more frequent in samples from Bolima community than other three communities.

## Conclusions

High frequencies of *kdrW* alleles and uneven frequencies of A296G *rdl* and *ace-1* in *An. gambiae* populations in Sierra Leone detected in the present study prompts the need for close vector monitoring of susceptibility levels to insecticides used in this country. It provides important information to the Sierra Leone Malaria Control Programme for development of insecticide resistance monitoring and adjusting the strategy to implement in this country.

## Data Availability Statement

The original contributions presented in the study are included in the article. Further inquiries can be directed to the corresponding authors.

## Ethics Statement

The project was informed to the study sites, and consent was obtained from the local authorities.

## Author Contributions

JY, SS, and NX conceived the study. FY and SS conducted sample collection and preparation. JY and FY performed the laboratory works. JY performed the result analysis. JY, CZ, SS, LW, and NX contributed to the study design. JY, SZ, HL, ZX, and NX contributed to experimental materials. JY drafted and revised the manuscript. All authors contributed to the article and approved the submitted version.

## Funding

This study was supported by the National Science and Technology Major Program of China (No. 2018ZX10101002–002), Technical Reserve Programme for Global Tropical Diseases Prevention and Control (No. 131031104000160004), the Fifth Round of Three-Year Public Health Action Plan of Shanghai (No. GWV-10.1-XK13), and Sierra Leone-China Second Phase of the Fixed Biological Safety Laboratory Technical Cooperation Project.

## Conflict of Interest

The authors declare that the research was conducted in the absence of any commercial or financial relationships that could be construed as a potential conflict of interest.

## Publisher’s Note

All claims expressed in this article are solely those of the authors and do not necessarily represent those of their affiliated organizations, or those of the publisher, the editors and the reviewers. Any product that may be evaluated in this article, or claim that may be made by its manufacturer, is not guaranteed or endorsed by the publisher.
